# Body circumferences: clinical implications emerging from a new geometric model

**DOI:** 10.1186/1743-7075-5-24

**Published:** 2008-10-06

**Authors:** Steven B Heymsfield, Allison Martin-Nguyen, Tung M Fong, Dympna Gallagher, Angelo Pietrobelli

**Affiliations:** 1 Merck & Company, Rahway, NJ, USA; 2 New York Obesity Research Center, St. Luke's-Roosevelt Hospital, Columbia University College of Physicians and Surgeons, NY, USA; 3 Pediatric Unit, Verona University Medical School, Verona, Italy

## Abstract

**Background:**

Body volume expands with the positive energy balance associated with the development of adult human obesity and this "growth" is captured by two widely used clinical metrics, waist circumference and body mass index (BMI). Empirical correlations between circumferences, BMI, and related body compartments are frequently reported but fail to provide an important common conceptual foundation that can be related to key clinical observations. A two-phase program was designed to fill this important gap: a geometric model linking body volume with circumferences and BMI was developed and validated in cross-sectional cohorts; and the model was applied to the evaluation of longitudinally monitored subjects during periods of voluntary weight loss. Concepts emerging from the developed model were then used to examine the relations between the evaluated clinical measures and body composition.

**Methods:**

Two groups of healthy adults (n = 494 and 1499) were included in the cross-sectional model development/testing phase and subjects in two previous weight loss studies were included in the longitudinal model evaluation phase. Five circumferences (arm, waist, hip, thigh, and calf; average of sum, C), height (H), BMI, body volume (V; underwater weighing), and the volumes of major body compartments (whole-body magnetic resonance imaging) were measured.

**Results:**

The evaluation of a humanoid geometric model based a cylinder confirmed that V derived from C and H was highly correlated with measured V [R^2 ^both males and females, 0.97; p < 0.001). Developed allometric models confirmed model predictions that C and BMI (represented as V/H) are directly linked as, C = (V/H)^0.5^. The scaling of individual circumferences to V/H varied, with waist the highest (V/H^~0.6^) and calf the lowest (V/H^~0.3^), indicating that the largest and smallest between-subject "growth" with greater body volume occurs in the abdominal area and lower extremities, respectively. A stepwise linear regression model including all five circumferences^2 ^showed that each contributed independently to V/H. These cross-sectional observations were generally confirmed by analysis of the two longitudinal weight loss studies. The scaling of circumference ratios (e.g., waist/hip) to V/H conformed to models developed on the scaling of individual circumferences to V/H, indicating their relations to BMI are predictable *a priori*. Waist, hip, and arm/calf circumferences had the highest associations with whole-body visceral adipose tissue, subcutaneous adipose tissue, and skeletal muscle volumes, respectively.

**Conclusion:**

These observations provide a simple geometric model relating circumferences with body size and composition, introduce a conceptual foundation explaining previous empirical observations, and reveal new clinical insights.

## Background

Body volume expands with the positive energy balance associated with adult human obesity. This enlargement of body volume is captured in two widely used obesity metrics, waist circumference and body mass index (BMI, weight/height^2^). Both measures are associated with total and regional adiposity [1-4] along with related biomarker abnormalities and clinical outcomes [5,6].

Circumferences, or more specifically perimeters, are relatively easy to measure and inexpensive to acquire in the clinical setting. Circumferences, applied either alone or in combination, in some cases have a higher predictive value of adverse outcomes than BMI [7,8]. Most recent randomized weight loss trials include longitudinal circumferential measurements, notably those of the waist and hip.

Circumferences and BMI similarly provide measures of total body and regional volumes, although at present a model that articulates and predicts the linkages between these measures is lacking. Specifically, how are circumference measurements related to body volume, weight, and BMI? Can a simple model be developed that predicts how these measures are associated with each other in the baseline state and following an intervention? Are there sex and age effects that moderate these relations? Answering these and related questions could provide important new insights into the interpretation and application of these widely applied measures of body size, shape, and health.

The aim of the present study was to develop and then evaluate a geometric model relating circumferential measurements with body volume and height.

## Methods

### Experimental design and rationale

#### Phase I

In the first phase of the study we developed a simple geometric model relating circumference measurements to body volume and height. We then applied the model to a group of healthy adults at or over the age of 18 years (Group I) as a means of exploring hypotheses generated during model development. Subjects in Group I had weight, circumference, and body volume measurements made on the same day at the New York Obesity Research Center (NYORC). Five representative circumferences were measured, arm, waist, hip, thigh, and calf.

The application of the model was then expanded in a larger sample of adults at or over the age of 18 years (Group II) to specifically develop a model-based prediction formula for waist circumference. The aim was to demonstrate the general applicability of the model, developed in the Group I cross-sectional cohort, with dynamic changes in body volume induced by weight loss interventions. Body volume was replaced in this portion of the study with body weight, making the assumption that the two are largely equivalent measures across a subject population with a uniform body density approximating 1.0 kg/L. The subjects in Group II included healthy adults evaluated across multiple earlier reported studies at the NYORC. The larger Group II database included subjects with a demographic profile similar to that of Group I who were also evaluated at the NYORC as part of multiple research programs.

#### Phase II

The hypotheses and prediction formulas developed in phase I were then evaluated by studying appropriate measures in subjects voluntarily losing weight in two supervised research programs [9,10]. Circumference measurements were made in these studies by trained observers under carefully controlled laboratory conditions. The two studies were as follows:

NYORC investigators evaluated adult overweight and obese subjects before and following a 12-week low calorie diet weight loss program [9]. The original study included two subject groups and subjects were pooled in the current study (Group III) that had complete sets of the five circumferences measured in Group I. Baseline and follow-up weight and circumference measurements were obtained in all subjects.

In the second study, Benedict and colleagues in 1915 reported the 31-day fluid-only total starvation experiment of Levanzin, a 40 year old normal weight male volunteer with a baseline BMI of 21.2 kg/m^2 ^[10]. Serial weight and circumference measurements (arm, waist, thigh, and calf) were reported for Subject L in the study publication.

The studies included in NYORC Groups I-III were all approved by the institutional review board and subjects signed informed consents prior to participation.

### Anthropometric measurements

Body weight and height in the three NYORC groups were measured in the morning after an overnight fast. Subjects in Groups I and III were evaluated in the same laboratory and subjects in Group II were evaluated in a second related NYORC laboratory. Although definitions of two anatomic circumference measurement sites were not identical, the absolute group values obtained did not differ significantly. Subjects wore a hospital gown and the circumference measurements were made by trained observers as follows:

Arm circumference was measured at a point equidistant between the acromion process of the right scapula and the olecranon process of the right ulna. The arm circumference was measured either with a Gulick II, tension-calibrated, tape measure (Groups I and III; Creative Engineering, Plymouth, Mich.) or a Dritz heavy-duty inelastic plastic fiber tape (Group III; Prym Consumer USA Inc., Spartanburg, SC) positioned perpendicular to the long axis of the right humerus with the arms hanging in a relaxed position by the side of the body.

Waist circumference was measured in Groups I and III at a point equidistant between the lowest costal border and the iliac crest at the mid-axillary line. The waist circumference was measured at the end of normal tidal expiration with the tape measure positioned perpendicular to the long axis of the body with the arms hanging relaxed at both sides of the body. Waist circumference was measured on Group III immediately below the end of the lowest rib.

Hip circumference was measured in Groups I and III at the level of the greater trochanter of the femoral bone that is palpated laterally and approximately coincides with the symphysis pubis level. Hip circumference was measured at the subject's dorsal side ensuring that the tape was maintained in a horizontal plane and the legs maintained in an adducted position without contraction of the gluteal muscles. The hip circumference in Group III was measured three-inches below the iliac crest.

Thigh circumference was measured at a level midway between the greater trochanter of the right femur and the most superior point on the lateral border of the right tibia that coincides with the popliteal fossa and patella level. The mid-thigh circumference was measured in the standing subject with the tape measure positioned perpendicular to the long axis of the right femur.

Calf circumference was measured at the level of maximum girth of the right calf from the lateral aspect of the leg with the subject standing in an elevated position on a stool. Calf circumference was measured by manipulating the tape in a series of up and down horizontal measurements so as to identify the maximal girth.

Some circumferences were unmeasured on occasion in several subjects and the sample sizes for each circumference evaluation are reported in the results. The anatomic locations of circumference measurements for Subject L were similar to those presented here with additional details provided in reference 10.

### Circumference model

**Figure 1:**
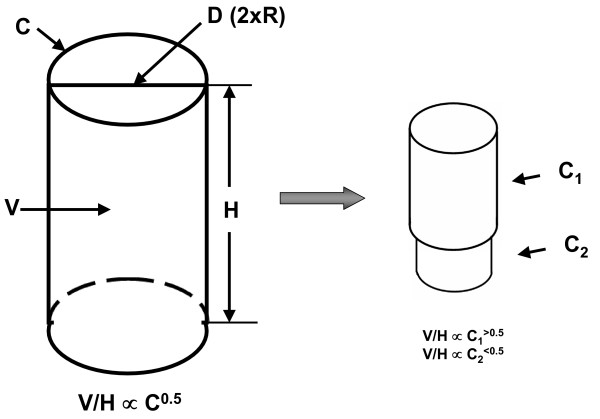
**Basic cylinder model (left) and additional hypothetical model with body volume (V) expansion including two different cylinders placed in series (right).** In the basic cylinder model and sphere, circumferences (C) as indicated in the figure are proportional (∝) to the square root of volume/height (V/H) (i.e., V/H^0.5^) and volume is proportional to the product of height and circumference squared. Cylinder 1 in the double cylinder model increases in volume more rapidly than cylinder 2 and this difference in the rate of expansion influences the scaling of total volume/height to the respective circumferences as indicated in the figure. Abbreviations: D, diameter; H, height; R, radius.

Although the body has an irregular shape, a simplifying assumption can be made that humans are cylindrical (Figure 1). According to this model, cylinder length is equivalent to height (H) and circumference is represented by perimeter measurements of the arm, waist, hip, thigh, and calf. Assuming for our initial model (Figure 1, left) that body "circumference (C)" is represented by each of the five designated perimeters, body radius (r) is equivalent to C/(2π). The cylinder's volume (V) can then be calculated as π(r)^2^H. Substituting circumference for radius in our model, we can solve for body volume as

(1) V = π(C/2π)^2^H

This model predicts that body volume or it counterpart, body weight, are directly linked to an integrated measure of body circumference and height. We can further transform equation 1 to reveal how the model relates to BMI,

(2) V/H = π(C/2π)^2^

and solving for circumferences,

(3) C = (4π(V/H))^0.5^

Our model then predicts that

(4) C ∝ (V/H)^0.5 ^

The symbol ∝ indicates "proportional to". In other words, assuming the body can be represented by a cylinder, circumference is proportional to the square root of volume per unit height. In practice body density in the general population approaches unity so that we can relate equation 3 to BMI by first considering the approximation C ∝ (W/H)^0.5^, with W as weight. Then, C ∝ (BMI × H)^0.5^, which is related to model 4.

In practice we are measuring 5 circumferences located across different body regions. With dynamic volume or closely-related body mass changes over time with aging or an intervention some regions may expand or contract more rapidly than others. For example, some portions of our model cylinder may increase more rapidly than others with volume gain and these regions may scale to volume/height with powers greater than 0.5. This modified model is shown in the right-hand portion of Figure 1. Likewise, when scaled to volume/height the slower growing regions may demonstrate powers of < 0.5. Our construct thus provides a framework for examining with empirical allometric models, described in the next section, how each circumference scales to volume/height and thus to define how different regions expand or contract with volume gain or loss, respectively. Body volume was measured in Group I subjects using underwater weighing as reported earlier [11].

### Allometric models

The scaling of circumferences to volume/height can be examined using the classic allometric model,

(5) Y = αX^β^ε

with Y dependent variable (i.e., circumference), X predictor variable (i.e., V/H), β the scaling exponent or power, α the proportionality constant, and ε the multiplicative error. When equation 3 is expressed in the form of natural logarithms, the scaling equation becomes

(6) log_e_Y = log_e_α + β log_e _X + log_e_ε

According to our basic cylinder model, β is equal to 0.5 in equation 3 for circumference (Y) scaled to volume/height (X).

A common practice is to express circumferences as ratios to one another.

The general allometric model summarized by equation 3 can be written individually for circumference 1 and circumference 2, both scaled to volume/height:

(7) C1 = α_1 _(V/H)^β1^

(8) C2 = α_2 _(V/H)^β2^

Therefore,

(9) C1/C2 = α_1/_α_2 _(V/H)^β1-β2 ^

When β1 and β2 are equal (i.e., when both circumferences scale the same to volume/height), the value of β1-β2 is 0 and a non-zero number raised to the power of zero equals 1. When the difference between β1 and β2 is at or near zero no correlation will be observed between the ratio of the two circumferences and volume/height. If the difference observed between β1 and β2 in equation 9 is not zero, the ratio of C1 to C2 will scale positively or negatively to volume/height.

The geometric circumference model was developed and explored in Group I using anthropometric data and body volume estimates measured by underwater weighing as reported earlier [11]. As the model was developed in the first phase of the study the results showed a large variation in the scaling of each circumference to volume/height. This finding implies that as the body "fills" with or loses fat during periods of energy imbalance there are large regional differences in the rates and amount of deposited or metabolized fat. This observation and related hypotheses prompted us to examine the associations between circumferential body dimensions and body composition, data available on the evaluated subject cohort [1]. Specifically, we carried out a simple descriptive analysis by correlating the five circumferences with total body, subcutaneous, and visceral adipose tissue (VAT) and total body skeletal muscle volumes as measured by whole-body magnetic resonance imaging [1,12].

### Statistical methods

Subject demographic characteristics are presented as the mean ± SD in tables and as the mean powers (± SEE) of scaling models in the figures. The statistical analyses were carried out using SPSS (SPSS for Windows, 11.5, SPSS Inc., Chicago, USA).

Volume calculated from circumferences and height as stated in equation 1 were regressed against measured total body volume based on underwater weighing using least squares multiple linear regression analysis. We additionally added age as a potential covariate to the sex-specific regression models. The average of the five measured circumferences was used as the circumference (C) term in equation 1.

The allometric model coefficients (i.e., α and β) were derived using least squares multiple linear regression analysis and log transformed data [1,13]. In some cases the dependent variables (i.e., circumferences) were also correlated with age and we therefore added age as an independent predictor variable when appropriate. These models were additionally explored for potential predictor variable interactions.

## Results

### Phase I cross-sectional studies

#### Baseline group characteristics

The baseline demographic information for Groups I and II is summarized in Table 1. There were 494 total subjects in Group I, 231 males and 263 females. The larger Group II had 1499 subjects, 479 males and 1020 females. The groups ranged in mean age from approximately 40 to 50 years and mean BMIs were all in the overweight range, ≥ 25 kg/m^2 ^and < 30 kg/m^2^. Both groups were ethnically mixed and reflected the local population demographics.

**Table 1:** Phase I subject characteristics.

	**Males**		**Females**	
**Group**	**I**	**II**	**I**	**II**

**N**	231	479	263	1020

**Age **(yrs)*	40.1 ± 14.1	45.7 ± 19.0	44.7 ± 16.1	49.6 ± 18.6

**Weight **(kg)	79.8 ± 12.8	79.3 ± 14.8	67.5 ± 15.4	73.6 ± 17.4

**Height **(cm)	176.7 ± 6.9	173.7 ± 8.0	161.8 ± 7.4	161.0 ± 7.4

**BMI **(kg/m^2^)*	25.5 ± 3.7	26.2 ± 4.4	25.8 ± 5.6	28.4 ± 4.4

Abbreviations: BMI, body mass index. Data are presented as group mean values ± SD.

#### Comparison of model-derived and measured body volumes

**Figure 2:**
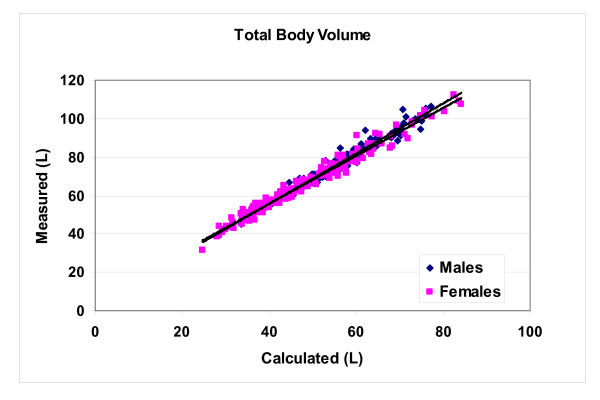
**Body volume (L) measured by hydrodensitometry plotted against body volume calculated using the simple cylinder model presented in equation 1.** The average of five measured circumferences represents C in equation 1. Males: Vmeasured = 1.30 × Vcalcluated+3.50; R^2^, 0.97; SEE 2.42) and females: Vmeasured = 1.25 × Vcalcluated+5.80; R^2^, 0.97; SEE 2.50).

Model-derived volume (equation 1) was significantly smaller (p < 0.001) but highly correlated (p < 0.001, Figure 2) with measured total body volume in the male (56.7 ± 9.9 vs. 77.5 ± 13.2 L; Vmeasured = 1.30 × Vcalcluated+3.50; R^2^, 0.97, SEE 2.42) and female (48.4 ± 12.6 vs. 66.1 ± 15.9 L; Vmeasured = 1.25 × Vcalcluated+5.80; R^2^, 0.97, SEE 2.50) subjects. Age was a non-significant predictor of measured body volume in both the male and female regression models.

#### Allometric analyses

**Figure 3:**
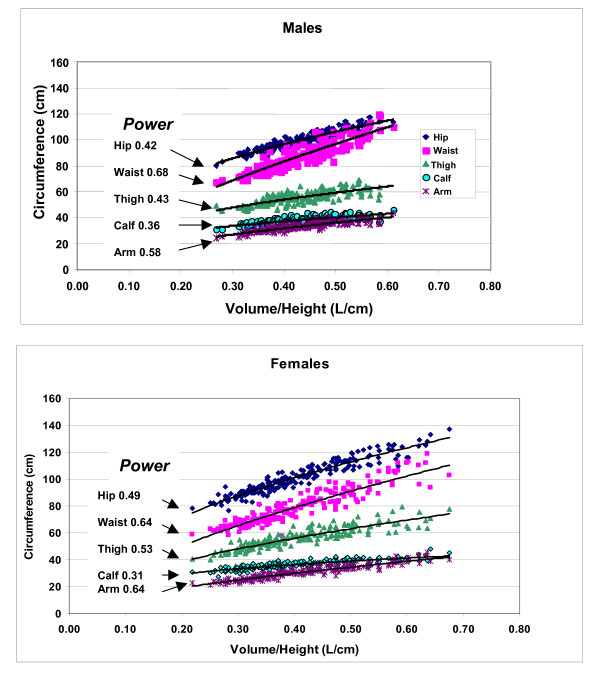
**Univariate allometric plots of circumferences versus volume/height for males (upper) and females (lower).** The respective powers of volume/height are shown in the figure; all models were statistically significant at p < 0.05.

The univariate plots of allometric relations examining the empirical associations between circumference measurements and volume/height are presented separately for males and females in Figure 3. The expanded multivariate models including age as a potential covariate are summarized in Table 2.

**Table 2:** Results of Group I scaling model development relating five measured circumferences to volume/height and age.

**Circumference**	**Males**						**Females**					
	
	**N**	**V/H**	**Age**	**Int**	**SEE**	**R**	**N**	**V/H**	**Age**	**Int**	**SEE**	**R**
**Arm**	159	0.61	-0.052	4.20	0.05	0.88	192	0.62	0.024	3.88	0.06	0.93

**Waist**	159	0.62	0.100	4.63	0.04	0.94	191	0.61	0.064	4.68	0.06	0.93

**Hip**	159	0.42	NS	4.96	0.03	0.94	190	0.49	NS	5.06	0.04	0.94

**Thigh**	150	0.50	-0.100	4.80	0.05	0.83	183	0.54	-0.032	4.65	0.06	0.88

**Calf**	155	0.38	-0.040	4.10	0.05	0.77	188	0.33	-0.046	4.07	0.05	0.83

**Average**	146	0.51	NS	4.57	0.02	0.98	176	0.54	NS	4.60	0.02	0.98

All models are p < 0.01. Circumferences and height (H) in cm, volume (V) in L, and age in yrs. Abbreviations: Int, intercept; R, correlation coefficient; SEE, standard error of the estimate. V/H and age are the beta weights and Int the intercept for a regression model of the form presented in equation 5.

The univariate plots shown in Figure 3 indicate that all five circumferences are strongly correlated (all p < 0.05) with volume/height and a consistent pattern is present across both males and females. First, the observed powers of volume/height ranged from ~0.3 to 0.6, not far from that predicted by the simple cylinder model estimate of 0.5 while consistent with a more complex regional model as depicted in the right-hand portion of Figure 1. Second, the observed powers of volume/height (V/H) for arm and waist circumferences were among the highest, hip and thigh intermediate, and calf the lowest for both males and females.

**Figure 4:**
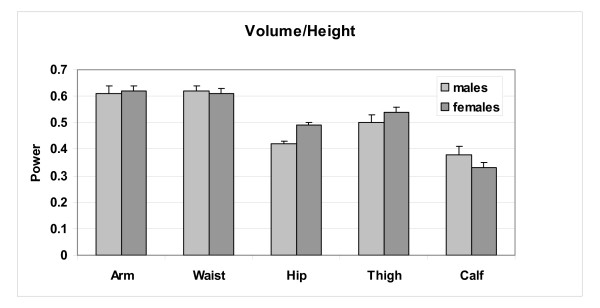
Power of volume/height (± SE) observed for each male and female multiple regression circumference prediction model presented in Table 2.

The analysis of the relations between circumferences and volume/height is expanded in Table 2 with multiple regression models. These models provide qualitatively similar scaling relations to those observed in Figure 3 and age entered as a predictor variable in all but the hip circumference models for both males and females. No interaction terms were detected in models including age as a predictor variable. Circumferences scale to volume/height with powers in these models as summarized in the table and as mean (± SE) values in Figure 4. The circumference scaling ranking (i.e., observed power of volume/height) is very similar in males and females, after adjusting for age:

*Arm & Waist > Thigh = ~0.5 > Hip > Calf*

Expressed as "growth", as subjects "enlarge" with greater volume/height across the group circumferential "expansion" rate is most rapid in the arm and waist areas, followed by the thigh, hip, and calf. A small difference is observed between males and females, with males "enlarging" at a greater rate around the waist and calf and females around the hips, thighs, and to a less extent the arms. This phenotypic description applies across weight stable subjects.

**Figure 5:**
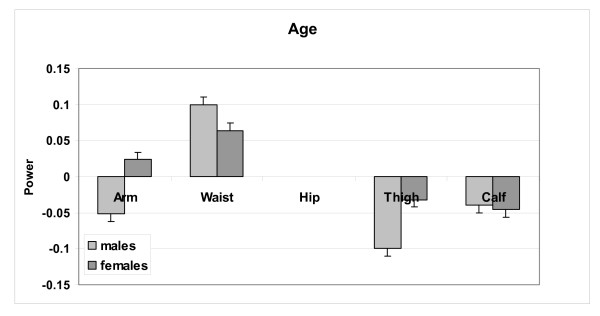
**Power of age (± SE) observed for each male and female multiple regression circumference prediction model presented in Table 2. **Age was not a significant predictor in the hip circumference prediction model.

When scaled to volume/height, some of the variation in all circumferences except hip could be accounted for by age. As shown in Table 2 and Figure 5, with greater age the effect on waist was positive and the effects on thigh and calf were negative for both males and females. The effect of age on arm circumference was also negative in males but positive in females. After adjusting for volume/height, older males and females thus had larger waist circumferences and smaller extremity circumferences with the exception of arm circumference in females.

In the next analysis the five measured circumferences were summed and their average was used to develop volume/height prediction models (Table 2). The male and female models were both highly significant (Rs = 0.98, p < 0.001). Neither male or female model included an age term and the circumference average scaled to volume/height with a power approaching 0.5 (males 0.51 and females 0.54). The male and female models were almost identical and, based on the average circumference, C ∝ (V/H)^0.5 ^and therefore V ∝ HC^2^.

As V ∝ HC^2 ^and thus V/H ∝ C^2^, we developed an expanded prediction model using stepwise multiple regression analysis in which each circumferece^2 ^was added as a potential independent predictor of volume/height. All five circumferences entered as independent variables in the models for males and females with high Rs (~0.98) and low SEEs (Table 3). For males, hip circumference entered that model first with an r-value of 0.932 followed by the addition of waist (0.956), arm (0.976), calf (0.981), and thigh (0.984) circumferences. For women, hip circumference also entered the model first (0.951), followed by the addition of arm (0.980), waist (0.984), thigh (0.987), and calf (0.988). Age was not a significant predictor variable in either model. Thus, hip circumference is the main predictor of volume/height in both men and women, with most of the remaining variance accounted for by waist in arm circumferences; thigh and calf circumference added minimally but significantly to the sex-specific models.

**Table 3:** Stepwise multiple regression model with volume/height (L/cm) as dependent variable and the five measured circumference^2 ^as predictor variables.

**Equation**								
**Subjects**	**Int.**	**Hip**	**Waist**	**Arm**	**Calf**	**Thigh**	**R**	**SEE**

**Males**	0.025	1.31E-05	1.47E-05	6.20E-05	4.00E-05	1.11E-05	0.98	0.013

	**Int.**	**Hip**	**Arm**	**Waist**	**Thigh**	**Calf**	**R**	**SEE**

**Females**	0.042	1.32E-05	7.14E-05	1.09E-05	1.64E-05	2.74E-05	0.99	0.015

Abbreviations: Int., intercept, SEE, standard error of the estimate. See text for circumference definitions. Both models are p < 0.001.

#### Circumference ratios

**Figure 6:**
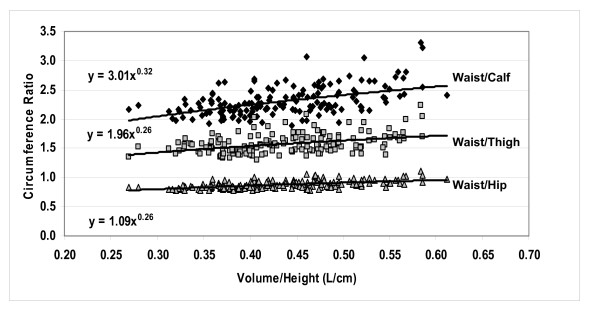
**Univariate plots of selected circumference ratios versus volume/height in males.** All of the correlations are significant at p < 0.05.

As defined by equation 9, circumference ratios will scale to volume/height as the difference between the respective circumference powers presented in Figure 3. For example, waist circumference scaled to volume/height in males with a power of 0.68 and hip scaled to volume/height with a power of 0.43. The actual waist/hip circumference ratio in males scaled positively to volume/height with a power of 0.26, approximately equal to the waist-hip circumference power difference of 0.25. Age added significantly to volume/height in the male waist/hip circumference prediction model. Thus, as total volume "expands" across a group of male subjects, waist circumference grows relatively more rapidly than hip circumference and the waist/hip circumference ratio predictably increases. Similarly, the waist/calf and waist/thigh circumference ratios scaled positively to volume/height with respective powers of 0.32 and 0.26 in males (Figure 6), nearly identical to that predicted from their univariate models presented in Figure 3 (i.e., 0.32, 0.25).

#### Group II prediction model development

The developed respective waist circumference (WC, cm) prediction models for males and females are

(10) log_e_WC = 4.59 + 0.11log_e _Age + 0.66 log_e_W/H

[R^2^, 0.86; SE, 0.05; p < 0.001]

and

(11) log_e_WC = 4.68 + 0.07log_e _Age + 0.64 log_e_W/H

[R^2^, 0.86; SE, 0.06; p < 0.001]

with weight (W) in kg, height (H) in cm, and age in years. These two models, neither of which included a significant interaction term, are similar to their volume-derived counterparts summarized in Table 2.

### Phase II longitudinal evaluations

The subject characteristics of Group III are presented in Table 4. At baseline there was no significant difference between measured and predicted (equations 9 and 10) waist circumference in Group III (X ± SD, 92.8 ± 9.8 cm vs. 92.2 ± 7.4 cm) and the two circumference estimates were highly correlated (R^2^, 0.75, p < 0.001).

**Table 4:** Phase II subject characteristics.

**Group III**	**Males**	**Females**	**Total**
**N**	11	65	76

**Age **(yrs)	41.1 ± 4.2	39.6 ± 7.5	39.9 ± 7.1

**Weight **(kg)	100.0 ± 12.1	82.0 ± 9.9	84.6 ± 12.0

**Height **(cm)	178.8 ± 8.2	162.3 ± 31.1	164.7 ± 8.4

**BMI **(kg/m^2^)	41.1 ± 4.2	39.6 ± 7.5	31.1 ± 2.8

**Weight Change **(kg)	-6.1 ± 3.2	-4.9 ± 3.3	-5.1 ± 3.3

Data are presented as group mean values ± SD.

There was a (X ± SD) 6.0 ± 3.9% weight loss over the 12 weeks in the group as a whole. Total body volume calculated from average circumference and height (equation 1) was highly correlated with body weight (i.e., a measure of body volume) at baseline and following weight loss (both R^2^, 0.93; p < 0.001). The change in calculated volume (4.6 ± 3.6 L) was also strongly correlated (R^2^, 0.71; p < 0.001) with the changes observed in body weight (4.9 ± 3.1 kg) over the 12 week study period.

Similarly, the weight/height predicted based upon individual circumference^2 ^values summarized in Table 3 was highly correlated with baseline and follow-up weight/height in males (r, 0.92 and 0.89, both p < 0.001) and females (r, 0.94 and 0.95, both p < 0.001). The actual (males, -0.033 ± 0.017; females, -0.030 ± 0.020 kg/cm) and predicted (-0.033 ± 0.017 and-0.029 ± 0.023 kg/cm) changes in weight/height observed over the 12 week period showed good absolute agreement and were highly correlated (pooled males and females; r, 0.80, p < 0.001).

**Figure 7:**
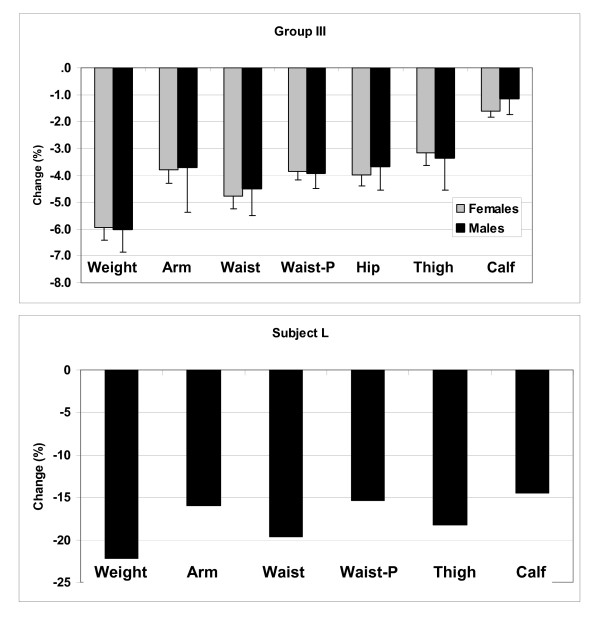
**Mean (± SE) percent change in body weight and circumferences in males and females participating in the Group III weight loss study (upper panel). **Percentage change in weight and circumferences observed after 31 days of total starvation in Subject L. Abbreviation: P, predicted.

The circumference changes, expressed as %Δ from baseline, are presented in Figure 7. The largest relative circumference reductions were observed for the waist (5.5%) and the smallest for calf (1.5%), with others intermediate, broadly consistent with the cross-sectional volume/height scaling models. There was a high correlation between the changes in measured and predicted waist circumference with weight loss (R^2^, 0.76, p < 0.001). The actual lowering of waist circumference with weight loss (5.5%) was greater than that predicted (4.9%).

Subject L, who weighed 61 kg at baseline, lost a total of 14 kg or 22.3% of baseline weight over the 31 day protocol. As shown in Figure 7, the largest corresponding circumference reduction was waist (19.6%) and the smallest calf (14.5%) with arm (15.9%) and thigh circumferences intermediate (18.2%). At baseline Subject L's measured and predicted waist circumferences were very similar (78.0 and 78.5 cm). Following weight loss, the measured change in waist circumference (15.3 cm) exceeded the predicted change (11.4 cm) by 3.9 cm.

#### Body composition associations

Of the five measured circumferences, waist scaled the highest and calf the lowest to volume/height. Similarly, with weight loss the largest relative reductions were observed for the waist circumference and the smallest for calf. These observations led us to hypothesize that variation in circumferential dimensions relative to volume/height are strongly linked to corresponding variation in adiposity, particularly adipose tissue distribution and muscularity.

**Figure 8:**
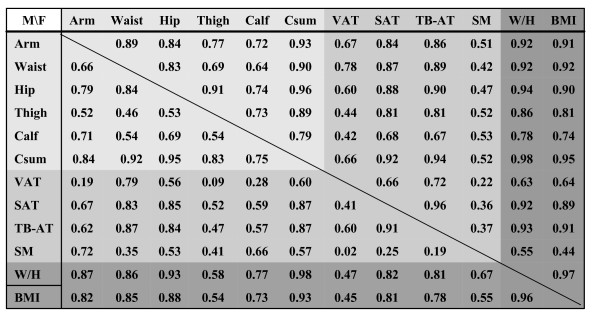
**Correlation matrix of Group I study measures.** Abbreviations: BMI, body mass index; Csum, sum of the 5 circumferences; SAT, VAT, TB-AT are subcutaneous, visceral, and total body adipose tissue volume; SM, skeletal muscle volume; W/H, weight to height ratio. N = 231 males and 263 females. All correlations r ≥ 0.20 are statistically significant at p < 0.05. Males comprise the lower half and females the upper half of the table as defined by the diagonal line. Shading represents grouping of measurement types (circumferences, body composition, and weight-stature).

**Figure 9:**
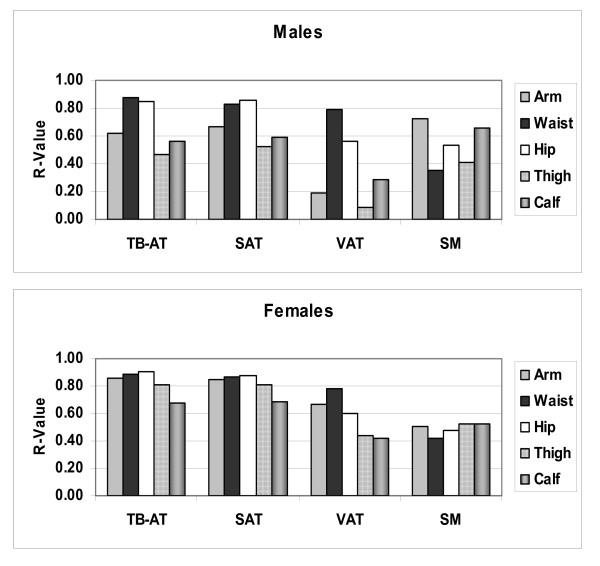
**Correlation coefficients (R-values) for simple linear regression analyses of five circumferences versus total body volumes of adipose tissue and skeletal muscle.** Abbreviations: SAT, TB-AT, VAT, subcutaneous, total, and visceral adipose tissue, SM, skeletal muscle.

The correlations (R-values) between each of the five measured circumferences and body composition are presented in Figure 8 and are summarized in Figure 9. Several observations are consistent across males and females. Of the five circumferences, waist was the strongest correlate of VAT volume and hip circumference was the strongest correlate of subcutaneous adipose tissue volume. Waist and hip circumferences where the highest and approximately equivalent correlates of total body adipose tissue volume. Extremity circumferences were the best predictors of skeletal muscle volume, arm in males and calf/thigh in females.

Since waist and hip circumferences were strongly correlated with VAT and subcutaneous adipose tissue (SAT), respectively, we hypothesized that the ratio of waist to hip circumference is well correlated with adipose tissue distribution. Accordingly, waist/hip was significantly correlated with VAT/SAT in males (r = 0.67) and females (r = 0.49), both p < 0.01. In contrast, waist and hip circumferences had weaker individual correlations with VAT/SAT in males (r = 0.54 and 0.24; both p < 0.01) and females (r = 0.40, p < 0.01; and p = 0.15, p = 0.05). The correlations between waist/hip ratio and VAT volume alone were weaker in males (r = 0.75) and females (r = 0.60) than those of waist circumference and VAT volume (r = 0.79 and 0.78)(all p < 0.01).

Body volume, expressed as weight/height, was strongly associated with total and subcutaneous adipose tissue volumes in males (r = 0.81 and 0.82) and females (r = 0.93 and 0.92, all p < 0.01) at approximately the same or higher level as the highest associating circumferences for those components. The correlations between waist circumference and VAT were greater than those observed for weight/height and visceral adipose tissue in both males and females. The sum of all five circumferences had similar magnitude correlations with body composition as weight/height and BMI.

## Discussion

The current study results strongly support the validity of a simple cylindrical model of the adult human body describing the relationships between circumferences, body volume, height, and weight. Our findings extend body geometric models first suggested by Bouchard and others [14-17] over one century ago that were aimed at providing estimates of surface area and other somatic physical characteristics. When the five representative circumferences were summed and averaged, the resulting estimated volumes and allometric functions generally conformed well to that predicted by the simple cylinder model. Moreover, multiple regression models based upon individual circumference^2 ^values were highly predictive of the changes observed in weight/height observed with diet-induced weight loss. Circumferences and BMI thus appear to reflect in common body volume according to simple geometric relations: V ∝ H × C^2^, C ∝ (V/H)^0.5^, and V/H × C^2^. Total body and subcutaneous adipose tissue, in turn, are large fractions of body volume (i.e., up to 50%) and hence strongly associate with the summed circumferences, weight/height, and BMI.

However, regional circumferences reflecting variation in body shape individually scaled to volume/height differently from that of the average of all five circumferences, suggesting that body segments "grow" across subjects at different rates with increasing or decreasing total body volume. Our observations, supported by limited longitudinal findings, indicate that the largest relative excursions with changes in body volume (i.e., weight) are present for waist circumference and the smallest for calf circumference. The other evaluated circumferences, arm, hip, and thigh are intermediate in their relations to volume/height between the waist and calf circumferences, with minor variation in the observed pattern between males and females. The individual circumferences, reflecting their regional volumes, had highly variable associations with adipose tissue and skeletal muscle component volumes. A fuller understanding of circumferential relations to obesity thus requires consideration of the more complex multiple interconnected cylinder model of the human body as outlined in Figure 1.

With changes in energy balance, the adult human body "grows" either by expansion with positive balance or by contraction with negative balance. Our findings suggest that, in the stable steady state, waist circumference scales the highest to volume/height across subjects in both males and females. This observation implies that with long-term changes in energy balance the structures encircled by the waist circumference expand or contract at a relatively greater rate than those of the other evaluated circumferences. Moreover, we confirmed this observation in the two evaluated longitudinal weight loss studies. In both of these studies, one of a single subject, the actual short term relative changes in waist circumference were even larger than predicted, suggesting that acute energy balance effects on body composition may differ from the pattern observed in weight stable subjects. Age moderated the relations between waist circumference and volume/height, with older subjects having a larger waist circumference than their younger counterparts.

The exploratory body composition studies suggest that, among the five evaluated circumferences, waist is the strongest correlate of VAT volume. This observation, in support of earlier studies [18-21], may account in part for the relatively large changes in waist circumference observed with weight loss, even larger than that predicted. Visceral adipose tissue appears to be a labile compartment, decreasing rapidly with induction of negative energy balance [22]. Moreover, in a recent study Mayer et al. [23] reported a greater increase in VAT than predicted with weight gain in patients recovering from anorexia nervosa.

The hip circumference scaled to volume/height intermediate (V/H powers = 0.42–0.49) between that of the waist circumference at the upper end (0.61–0.62) and the calf circumference at the lower end (0.33–0.38). Similarly, the hip circumference changes with weight loss in the longitudinally evaluated subjects were intermediate between those of the waist and calf circumferences. The hip circumference was the strongest correlate among the five evaluated circumferences of subcutaneous adipose tissue volume in males and females. Both waist and hip circumferences were approximately equivalent in their strong correlations with total body adipose tissue volume.

Unlike waist and hip circumferences, the calf circumference scaled relatively weakly to volume/height and had the smallest relative changes with weight loss in longitudinally evaluated subjects. Moreover, the calf circumference was smaller in older subjects after adjusting first for volume/height. This observation is consistent with the finding that of the five evaluated circumferences, calf circumference was among the strongest correlates of total body skeletal muscle volume and there is a loss of skeletal muscle mass with aging [24].

Arm circumference scaled high to volume/height with powers the same (0.61–0.62) as those of waist circumference. However, with weight loss, relative arm circumference changes were less than those of the waist and similar to those of the other intermediate scaling circumferences hip and thigh. Arm and thigh circumferences were also smaller with greater age, after adjusting for volume/height, except for arm circumference in females which scaled positive with greater age. Collectively, all three extremity circumferences (i.e., arm, calf, and thigh) are thus smaller with greater age with the exception of arm in females. The extremity circumferences were all strongly correlated with total body skeletal muscle mass, which likely accounts for their smaller size with greater age, after adjusting for volume/height.

In addition to individual circumferences, ratios are widely used in the area of clinical obesity evaluation [25]. Our observations show that circumference ratios scale to volume/height almost identical to that predicted from the scaling of individual circumferences to volume/height. This finding creates the possibility of developing several different circumference ratios with their relationships to body size and body composition predictable *a priori*.

The ratio of waist to hip circumference is the most widely used in current obesity evaluations, and the present study results suggest that this phenotypic measure provides an index of the amount of VAT relative to subcutaneous or total adipose tissue volumes. Our results indicate that the waist to hip circumference ratio increases as a function of volume/height and age, with the ratio larger in males than females. As a measure of fat partitioning, the waist to hip circumference ratio is likely strongly influenced by hormonal and related genetic mechanisms. In contrast, waist and hip circumferences by themselves are highly correlated with VAT and SAT volumes (Figure 8), but provide lower magnitude correlations alone with VAT/SAT than the waist/hip circumference ratio. Our results also indicate that the correlations between waist to hip circumference ratio and VAT volume were less than those of waist circumference alone.

## Clinical implications

The following set of linkages describes the role of body circumferences in nutritional assessment evaluations:

Circumferences→Body Composition→Biomarker Effects→Clinical Disease→Outcomes

**Figure 10:**
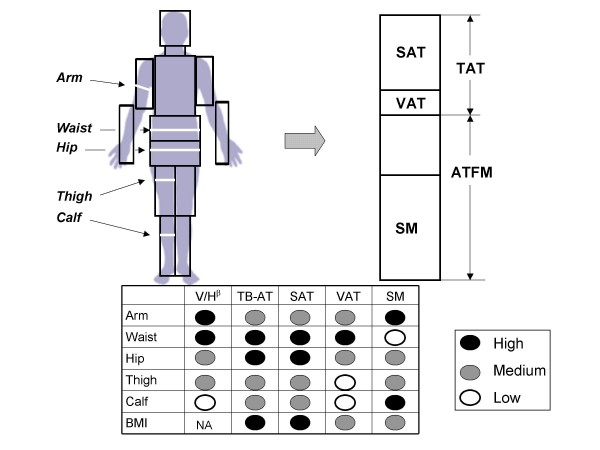
**The potential utility of circumference measurements in providing surrogate estimates of body compartments.** The body is segmented into cylinders with some including an associated circumference. The cylinder circumferences and volumes relate to body compartments with correlations ranging from low to high as indicated in the figure. The circumferences also scale to volume/height (V/H) with powers (β) of variable magnitude, reflecting their respective sensitivities to body "growth" as defined by between-subject differences. Abbreviations: BMI, body mass index; NA, not applicable; SAT, VAT, and TB-AT, subcutaneous, visceral and total body adipose tissue; SM, skeletal muscle.

Many reports document these various associations and hence the clinical value of body circumferences and their ratios. In the present study we focused specifically only on the first two steps in this set of relations and a qualitative composite summary of our cross-sectional findings is presented in Figure 10. Of the evaluated circumferences, the waist uniformly scales among the highest to body volume and has the strongest associations with adipose tissue components. Waist circumference also appears particularly sensitive to changes in energy balance with acute weight loss interventions. Calf circumference is only weakly associated with body volume and is minimally influenced by body volume changes while maintaining strong associations with skeletal muscle mass across weight stable subjects.

Body weight and height are fundamental clinical measurements and BMI provides a composite measure of body volume and total body adiposity. Thus, from the perspective of the current study, waist circumference and BMI would appear complementary clinical measurements, waist circumference capable of detecting more individual variation in VAT and changes in energy balance with BMI capturing more individual variation in skeletal muscle, and presumably lean mass. While these differences between the two measures may be subtle, they might in part explain why in some studies BMI and waist circumference are additive in predicting biomarker effects and outcomes [26-29].

Circumference ratios are surrogates of regional and total body composition ratios that in turn reflect mechanistic hormonal and metabolic processes. The waist to hip ratio would not only provide a measure of adipose tissue distribution but may capture some individual variation in skeletal muscle and lean mass as well. Also, combining individual or multiple circumference measurements with BMI will provide additional body composition information as approximated by examining relations described in the figure. By combining circumferences with each other and potentially BMI, it should be possible to construct an individual's "somatogram", their unique shape relative to the general population [15-17].

Since we have now shown that waist circumference and BMI are linked through their common relations to body volume, is there any value in measuring both or even additional circumferences with weight loss? With usual diet-induced weight loss all of these measures will change in parallel through their associations with changes in body volume, although the possibility exists that variation will be observed within individuals or secondary to the selective treatment actions on adipose tissue or skeletal muscle tissues. Moreover, combining two or more measures may capture even greater individual variation or group responses to treatment. Our stepwise multiple regression models presented in Table 3 suggest that changes in weight with dieting reflect the sum of independent sex-specific changes in regional volumes as represented by all five measured circumferences. An important need exists for future carefully controlled studies that include multiple circumferential and related body composition measurements.

Our focus in this report was on body circumferences as measured by anthropometric methods. A more fruitful approach, beyond the scope of the present study, would be to link easily measured body compartments such as total body water, fat mass, and fat-free mass with clinically-relevant BMI-body volume models.

## Conclusion

Our approach, the first of its type that we are aware of, was stimulated by the widespread use of and discussions surrounding circumferences and BMI as measures of adiposity and related health risks [19]. Our focus was to clarify, using simple geometric models, how circumferences and BMI are related to one another in the baseline state and with interventions that modify energy balance. Circumferences and BMI can be envisioned as different measures of the same body volume system described conceptually as *V ∝ H × C*^2^, *C ∝ (V/H)*^*0.5*^, and *V/H ∝ C*^2^. Each circumference in turn deviates from this central model providing unique body composition and energy balance information. These fundamental relations provide a basis for understanding and applying circumferences in research studies and clinical practice.

## Abbreviations

BMI: body mass index; C: circumference; H: height; NYORC: New York Obesity Research Center; SAT: subcutaneous adipose tissue; V: volume; VAT: visceral adipose tissue; W: weight.

## Competing interests

SBH, AMN, and TMF are employees of Merck & Co. DG and AP have no financial or personal conflicts of interest.

## Authors' contributions

SBH was the Principal Investigator and was responsible for design, data collection, analysis, and article preparation. AMN was responsible for analysis, and article preparation. TMF was responsible for analysis, and article preparation. DG was responsible for data collection, analysis, and article preparation. AP was responsible for design, analysis, and article preparation.
